# Three-Year Outcomes of Wet Age-Related Macular Degeneration Treatment in Polish Therapeutic Programs

**DOI:** 10.3390/medicina58010042

**Published:** 2021-12-27

**Authors:** Małgorzata Figurska, Marek Rękas

**Affiliations:** Department of Ophthalmology, Military Institute of Medicine, The Central Clinical Hospital of the Ministry of National Defense, 04-141 Warsaw, Poland; mrekas@wim.mil.pl

**Keywords:** wet age-related macular degeneration, intravitreal injections, aflibercept, ranibizumab, electronic registry

## Abstract

*Background and Objectives*: Wet age-related macular degeneration (wAMD) is a chronic, progressive disease of the central part of the retina. Standard treatment for wAMD consists of multiple intravitreal injections of anti-vascular endothelial growth factor drugs. The study goal was to evaluate the three-year effectiveness of wAMD treatment with aflibercept and ranibizumab as part of the therapeutic program in routine clinical practice. *Materials and Methods*: 1430 patients (possessing 1430 wAMD eyes) with median age of 78.0 years (71.0, 83.0) were enrolled in a non-randomized, retrospective, observational, multicenter study; 804 (56.2%) eyes were treatment-naïve. Therapy was carried out in accordance with the guidelines of the treatment program (the fixed or *pro re nata* regimen). *Results*: After the first year of treatment, there was a gain of 2.03 (12.15) letters; after the second, 0.94 (13.72) (*p* ˂ 0.001); and after the third, 0.17 (14.05) (*p* ˂ 0.001). There was a significant reduction in the central retinal thickness. In the first year, the patients received 7.00 (5.00, 8.00) injections. In the following years, a significantly lower number of injections (4.00 (2.00, 5.00)) was administered. After the first year, there was a significant difference in the distribution of the best corrected visual acuity according to the Early Treatment Diabetic Retinopathy Study protocol, with more frequent values in the ranges > 35 ≤ 70 for this parameter and > 70 letters in the treatment naïve eye subgroup. After the first year, central retinal thickness in treatment-naïve eyes was significantly reduced. *Conclusions*: Regular treatment of wet age-related macular degeneration as part of the treatment program achieves functional stabilization and significant morphological improvement over a long-term, three-year follow-up, with significantly fewer injections needed after the first year of treatment.

## 1. Introduction

Since autumn 2015, wet age-related macular degeneration (wAMD) has been treated in Poland as part of the treatment program (TP). Currently, there are over 28,000 patients undergoing therapy, and more than 70,000 applications are registered in the electronic Therapeutic Program Monitoring System (TPMS) [1,2,3].

wAMD is a chronic, progressive disease of the central part of the retina that affects people over the age of 45. A patient with wAMD requires many years of monitoring and treatment. The current, widely-accepted therapy for wAMD consists of repeated intravitreal injections of drugs blocking vascular endothelial growth factor (VEGF) [4]. As part of the TP, therapy with the nonselective VEGF inhibitors ranibizumab (Lucentis, Genentech/Novartis) and aflibercept (Eylea, Regeneron Pharmaceuticals/Bayer), which additionally blocks placental growth factor (PlGF), is conducted [5,6,7,8,9]. From November 1 2021, brolucizumab (Beovu^®^/Novartis) has also become available.

Therapy of wAMD with repeated intravitreal injections of anti-VEGF agents is chal-lenging in terms of procedure organization. In developed countries, prolonged mean life-time results in increasing number of people affected with wAMD. Efforts are made to pro-vide patients with systematic monitoring of disease activity and treatment via therapeutic programmes, which enable improvement and long-term maintenance of functional pa-rameters.

The aim of this study was to evaluate the effectiveness of treating wAMD with intravitreal injections of anti-VEGF drugs, carried out in routine clinical practice in the Polish treatment program, over a period of three years.

### 1.1. Materials

This was a non-randomized, retrospective, observational, multicenter (20 wards) study of eyes qualified for treatment in the TP due to wAMD. It was conducted in the Mazovia region in the period between January 2016 and June 2017. The study used anonymous data contained in the TPMS, supervised by the National Health Fund (NHF) and the Minister of Health. The anonymous analysis of the TPMS database was approved by the President of the National Health Fund and the Bioethics Committee of the Military Institute of Medicine (decision number 68/WIM/2017).

In the study, as in the TP, the following criteria were in force: (1) presence of active classic, occult or mixed subretinal neovascularization, occupying more than 50% of the lesion, confirmed in optical coherence tomography (OCT) and fluorescein angiography (FA) or angio-OCT; (2) patient age over 45; (3) lesion size less than 12 DA (disc area); (4) best corrected visual acuity (BCVA) in the treated eye of 0.1–0.8, determined according to the Snellen chart (or Early Treatment Diabetic Retinopathy Study (ETDRS) equivalent), although from January 2017 the lower limit of qualification was BCVA 0.2; (5) consent of the patient to perform intravitreal injections; (6) no dominant geographic atrophy; (7) no dominant hemorrhage; 8) from January 2017 no significant permanent damage to the foveal structure (defined as fibrosis, atrophy or disciform scar). For the purposes of the study, the data on the BCVA value measured using the Snellen chart were converted into numbers of ETDRS letters [10].

Each eye was consecutively assigned a number according to the order of system registration. Consent to treatment in the drug programme was given by the Coordinating Expert Group after review of anonymized application submitted to TPMS by managing physician. Coordinating Expert Group evaluated OCT images attached to the application in terms of disease activity, i.e., presence of subretinal, intraretinal, and subretinal pigment epithelium fluid spaces. During the qualifying examination and in the course of treatment, respective fluid spaces were not individually recorded. Eyes without signs of neovascularization (i.e., with no fluid spaces) and with dominant, irreversible macular changes described above were not eligible.

The exclusion criteria (as specified in the drug programme) were as follows: (1) hypersensitivity to the drug or excipient; (2) active infection of the eye or its surroundings; (3) active severe endophthalmitis; (4) pregnancy or breastfeeding; (5) drug-related side effects that prevent its further use; (6) rhegmatogenous retinal detachment or stage 3 or 4 macular hole; (7) disease progression defined as: (a) BCVA deterioration by ≥ 30 letters according to the ETDRS chart (or the Snellen chart equivalent), lasting longer than two months; (b) BCVA deterioration to ≤ 0.05 determined according to the Snellen chart (or the ETDRS equivalent), persisting for more than two months; or (c) BCVA deterioration to < 0.2 according to the Snellen chart (or the ETDRS equivalent) from January 2017, persisting for more than two months; (8) from January 2017 permanent damage to the structure of the fovea (fibrosis, atrophy or disciform scar).

### 1.2. Methods

The recommended dose of aflibercept was 2 mg per intravitreal injection. In the first year, treatment-naïve eyes were treated according to the established schedule, in accordance with the VIEW (VEGF Trap-Eye: Investigation of Efficacy and Safety in Wet AMD) protocol. There was a saturation phase (one injection per month for three consecutive months), and then the drug was administered every two months. In the subgroup of eyes in which therapy continued, aflibercept was dosed according to the *pro re nata* (PRN) regimen, i.e., in case of relapse (occurring of new fluid spaces or enlarging of previously present ones with accompanying visual acuity deterioration).

According to the TP, control visits had to take place no less frequently than every two months.

The recommended dose of ranibizumab was 0.5 mg per intravitreal injection. The drug was administered at monthly intervals until disease activity ceased and the BCVA was stabilized. Relapse of disease activity (occurring of new fluid spaces or enlarging of previously present ones) and/or BCVA deterioration were indications for reinjection of ranibizumab. Control visits had to take place no less frequently than every two months.

The functional parameter BCVA and the morphological parameter CRT were analyzed statistically. The final results obtained were compared with the baseline values in search of statistically significant differences. An analogous analysis was performed in treatment-naïve and continuing-treatment subgroups and compared against the number of follow-up visits and anti-VEGF injections.

Statistical analysis used typical measures of location and dispersion. The normality of the distribution of individual variables was tested with the Shapiro–Wilk test. In the case of normal distribution, the mean value and standard deviation (SD) were reported. In the case of a non-parametric distribution of the variables, the median and the interquartile range (IQR) (i.e., the 25th and 75th percentiles or Q1 and Q3) were provided. Categorical variables were compared using the Fisher or Chi-square test. The values of variables with normal distribution were compared with Student’s *t*-test or ANOVA (for more than two values). The U-Mann–Whitney or Kruskal–Wallis test was used to compare the median value. The graphs present the 95% confidence interval for the medians.

*p*-values below 0.05 were considered significant. Two independent tests were performed to confirm the result. The analysis was performed using the StatSoft statistical software, version 3.4.0 (TIBCO Software Inc, Hamburg, Germany).

## 2. Results

### 2.1. Baseline Data

To assess the outcomes of the three-year treatment of wAMD in the Mazovia region, a group of 1430 eyes (in 1430 patients) was selected. Two subgroups of patients (eyes) were formed based on the criterion of the time of initiation of wAMD treatment; 804 (56.2%) eyes were treatment-naïve (previously untreated), and 626 (43.8%) eyes were continuing treatment started outside the TP. The baseline demographic data (V0 qualifying visit) of the study group are presented in Table 1. The median time of prior treatment (from disease diagnosis to joining the TP) in the subgroup continuing treatment was 400.5 (102.75, 954.75) days, and the number of anti-VEGF injections (aflibercept, ranibizumab, bevacizumab) in this period was 4.0 (3.0, 6.0). The median age of patients was 78.0 (71.0, 83.0) years. Women constituted 64.2% of the study group. The median baseline BCVA was 65.10 (50.05, 73.91) ETDRS letters (0.4 (0.2, 0.6) according to the Snellen chart). There was no significant clinical difference in baseline BCVA values according to ETDRS and Snellen charts between the subgroups (with an existing statistical difference). There was a signifi-cant difference in the BCVA distribution according to ETDRS. In treatment-naïve eyes, BCVA was found more frequently in the ranges > 35 ≤ 70 and > 70 ETDRS letters. There was no significant difference in the distribution of BCVA according to the Snellen chart, as well as in the percentage share of active leakage in the lesion, central retinal thickness (CRT), and percentage distribution of its value between subgroups. The significance of the distribution of wAMD forms between the subgroups was borderline, with more frequent occult and mixed forms. Aflibercept was administered to 1077 (75.3%) eyes. The others were treated with ranibizumab.

### 2.2. Functional and Morphological Outcomes

All enrolled patients completed the first year of treatment, 1215 (85% of the initial group) completed the second year of treatment, and 992 (69.4% of the initial group) completed the third year. The reasons for the reduction in the number of patients in the following years were: death, lack of cooperation between the patient and the doctor, the occurrence of exclusion criteria in the form of irreversible changes in the fovea due to scar-ring, and atrophy. The *p*-values were always calculated within groups of the same size, i.e., for the first year, groups of 1430 were compared; while for the second year, the results for 1215 subjects were compared with the data of these patients in V0. Similarly, after the third year, the results for 992 patients were compared to their baseline data.

**Figure 1:**
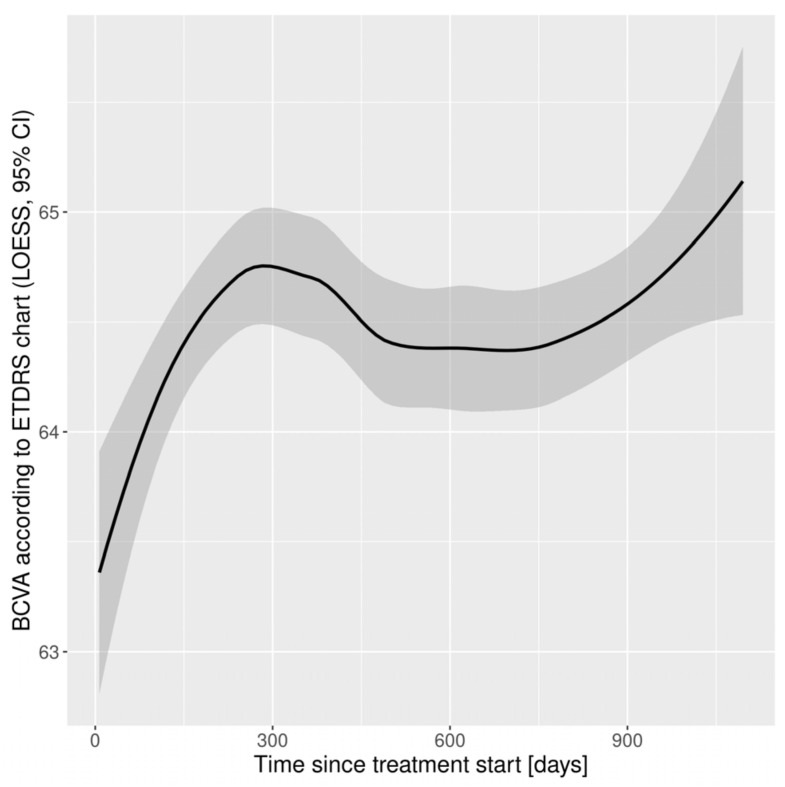
Functional outcomes: changes in Best Corrected Visual Acuity according to ETDRS chart in study population. BCVA, Best Corrected Visual Acuity; CI, Confidence Interval; ETDRS, Early Treatment Diabetic Retinopathy Study; LOESS, Locally Estimated Scatterplot Smoothing.

**Figure 2:**
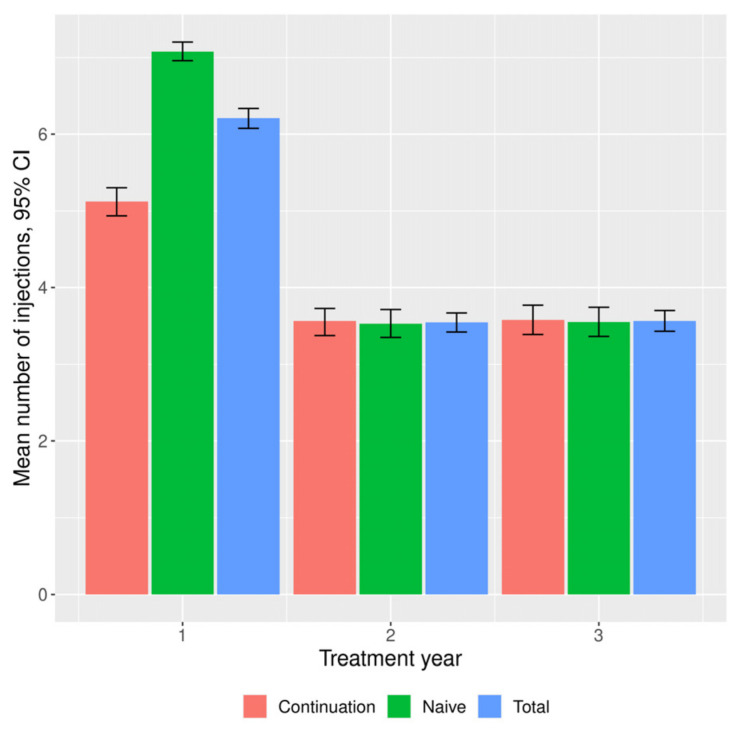
Mean numbers of injections during the study period in general study population and in continuation and naïve subgroups. CI, Confidence Interval.

The functional and morphological outcomes are presented in Table 2 and Table 3, respectively. The median BCVA expressed in ETDRS letters and according to Snellen did not change clinically significantly, but there was a statistically significant difference (*p* < 0.05) after the first and the second year. After the first year, patients had gained on average 2.03 (12.15) ETDRS letters. In the following years they gained significantly fewer letters: 0.94 (13.72) after the second year, and 0.17 (14.05) after the third year. According to the Snellen chart, no clinically significant changes in BCVA were noted. The functional outcomes are shown in Figure 1. A significant reduction in CRT from baseline was maintained in the following years (Table 3). It was accompanied by a significant change in the value of the parameter, which was maintained in the following years. In the second and third year, significantly fewer injections were made than in the first year (4.00 (2.00, 5.00) versus 7.00 (5.00, 8.00) injections, respectively) (Table 3, Figure 2). In the first year, there were 9.00 (9.00, 10.00) visits per patient; in subsequent years significantly fewer visits took place (7.00 (6.00, 8.00)).

### 2.3. Continuation and Naive Subgroup Comparison

**Figure 3:**
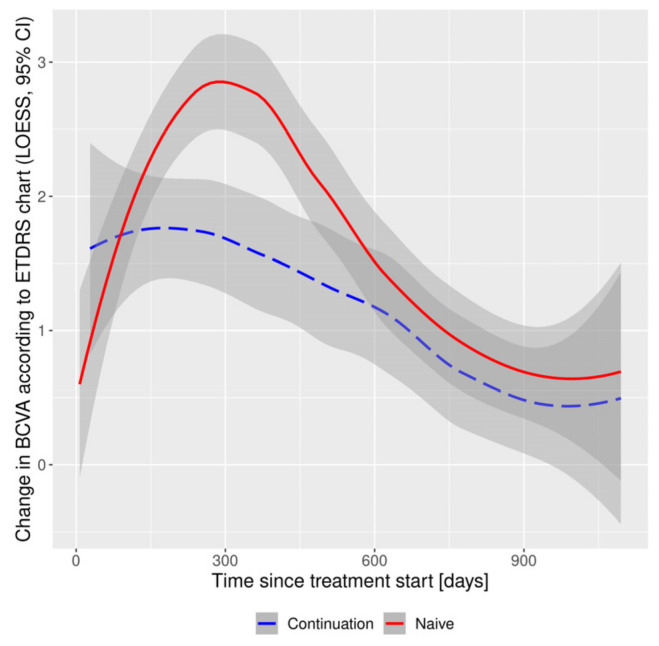
Functional outcomes: changes in Best Corrected Visual Acuity according to ETDRS chart in continuation and naïve subgroups. BCVA, Best Corrected Visual Acuity; CI, Confidence Interval; ETDRS, Early Treatment Diabetic Retinopathy Study; LOESS, Locally Estimated Scatterplot Smoothing.

There were no significant differences between the subgroups in the number of ETDRS letters gained in subsequent time intervals (Figure 3) or in the number of patients gaining ≥ 10 ETDRS letters. By the end of year 1, a significant difference in the distribution of BCVA according to ETDRS was maintained, with more frequent BCVA in the ranges > 35 ≤ 70 and > 70 ETDRS letters in the treatment-naïve subgroup. There was a significant reduction in CRT from baseline in the subgroups at all time intervals. After year 1, CRT in treatment-naïve eyes was significantly lower compared to the subgroup continuing treatment, as was the reduction in the parameter size. In year 1, significantly more injections were performed in the treatment-naïve subgroup (7.00 (7.00, 8.00) vs. 5.00 (3.00, 7.00) in a group continuing therapy). There was no such relationship in the following years. In both subgroups, the number of injections significantly decreased (to 4.00 (2.00, 5.00)) in the following years, as did the number of follow-up visits.

There were no adverse effects related to the intravitreal administration of drugs in the TP in the study period.

## 3. Discussion

In developed countries, it has become a standard to collect data on the effectiveness of wAMD treatment in electronic databases. Our observation was also based on the analysis of data from the TPMS. The analysis covered a large group of more than 1400 patients from the central region, one of the most populous regions of Poland. In year 1 of treatment, a mean gain of 2.03 (SD 12.15) ETDRS letters was achieved, with a median of 7.00 (5.00, 8.00) anti-VEGF injections. After the third year, the number of letters gained was significantly lower and amounted to 0.17 (SD 14.05), with a significantly lower number of 4.00 (2.00, 5.00) injections. In the presented analysis, 75% of patients were treated with aflibercept. Therefore, in year 1, most treatment-naïve eyes were treated according to a fixed schedule of three saturation doses, followed by an aflibercept injection every two months. During the three-year period, no cases of switching to another drug within the TP were reported.

The first-year functional outcomes are comparable to others already published based on data from the TP. In the one-year, multicenter follow-up of 2718 patients treated for wAMD within the TP across Poland, an average gain of 2.9 ETDRS letters in treatment-naïve eyes was noted, with a similar median of 7 injections [2]. A significant morphological improvement with a reduction in CRT was observed, similar to the material analysed in this study.

The results obtained after the first year of treatment in the TP in the Mazovia region are similar to the observations of other researchers. Talks et al. [11] reported a gain of 5.1 letters after an average of 7 injections in the group of 1840 treatment-naïve eyes treated according to the VIEW protocol. In the multicenter German PERSEUS study, with regular treatment with aflibercept, an improvement in BCVA was achieved from + 3.1 ± 10.7 ETDRS letters (in the previously-treated eyes subgroup) to + 8.0 ± 17.7 (in the treatment-naïve eyes subgroup) [12]. Based on the results of the PERSEUS study, Watchlin et al. emphasized a significantly greater positive effect on the functional outcome of treatment regularity compared to the number of injections ≥ 7 in the first year, but administered inconsistently with the VIEW protocol [13]. In our study, at the end of year 1, no significant differences were found in the number of ETDRS letters gained, with a significantly lower number of injections in the subgroup continuing the therapy. On the other hand, Almuhtaseb et al. [14] presented the annual results of regular aflibercept administration in wAMD in a group of 255 treatment-naïve eyes (in 223 patients) in England. BCVA improved by an average of 8 letters, which was again comparable to the results of the VIEW 1 and VIEW 2 studies.

In a 2-year study based on the daily clinical practice of wAMD therapy with aflibercept in France (4-year observational RAINBOW study), Weber et al. [15] achieved improvement in BCVA by an average of + 3 ETDRS letters (gain of 0.94 ETDRS letters in our observation after the second year of treatment).

In the TP, the principle of established, regular evaluations is adopted. Injections in subsequent years of treatment are performed according to the PRN regimen, i.e., in the case of relapse of disease activity with deterioration of BCVA and/or morphological changes. In the RAINBOW study, a mean loss of 2.5 ETDRS letters was reported after two years in the subgroup treated irregularly with aflibercept. In our observation, no worsening of BCVA was found in any of the subgroups over the three-year period. In the RAINBOW study, in the first year of treatment, an average of 6.0 injections (median of 7.0 in our observation), and 8.8 injections in two years (median of 4.00 injections in subsequent years in our observation) were performed. The numbers of injections, both in the RAINBOW study and in our study, are similar to the VIEW study. Weber et al. emphasize the importance of the saturation phase and regularity of wAMD therapy to achieve significantly better and lasting functional effects. This recommendation is met by the Polish TP and our study.

Eter et al. [16] presented the 2-year results of the German PERSEUS study. Eyes continuing the therapy, treated regularly, gained an average of 0.8 ETDRS letters (0.69 ETDRS letters in our observation), and the irregularly treated eyes lost on average 2.7 letters (in our observation there was no deterioration in BCVA). On average, in the entire study group, the eyes gained 1.1 ETDRS letters (0.94 in our material). Unfortunately, Eter et al. found that more than 80% of patients were treated irregularly. Irregularly-treated eyes received an average of 7.8 (± 4.09) injections of aflibercept, while regularly-treated eyes received significantly more (13.1 (± 1.3)) over the period of two years. Eter et al.’s observations indicate that the best functional effects are obtained with regular visits and injections. After two years, in the PERSEUS study, the best functional outcomes were seen in the treatment-naïve eyes treated in the first year according to the VIEW protocol and in the second year receiving ≥ 4 injections of aflibercept. In our observation, a significant difference in the distribution of BCVA according to ETDRS was maintained until the end of the first year, with more frequent BCVA in the ranges > 35 ≤ 70 and > 70 ETDRS letters in the treatment-naïve subgroup. Moreover, after the first year, CRT in treatment-naïve eyes was significantly lower than in the group continuing treatment, as was the value of the reduction in the size of the parameter.

Eleftheriadou et al. [17] published the results of a 3-year wAMD treatment with aflibercept according to data from the Moorfields Eye Hospital electronic database; 108 eyes (102 patients) have completed the third year of follow-up. The investigators noted a reduction in CRT of 77.9 ± 101.4 µm, comparable to our observation of −65.00 (−142.50, 7.00) µm at the end of year 3. The mean number of injections after three years was 15.9 ± 6.1 (comparable with our observation). The long-term study by Eleftheriadou et al. once again confirmed the good and stable (as in our observation) functional and morphological effects of regular wAMD therapy.

Although the vast majority of patients in our study were treated with aflibercept, it is worth quoting data on ranibizumab therapy. According to a report from the United Kingdom, treatment with ranibizumab in daily practice with a saturation phase followed by PRN regimen had a functional effect of + 2.0 ETDRS letters after one year, + 1.0 after two years and −2.0 after three years [18]. The number of injections in the following years is noteworthy: 5.4 in the first year, and 4 in the following years, with a significant number of 9–8 follow-up visits according to the report. In our observation, better functional results, a greater number of injections in the first year and a significant decrease in the number of visits in the following years were achieved. This means that the regulated treatment of wAMD in the TP, primarily with aflibercept, translates into good long-term functional effects.

Results for 5–10 years of treatment of wAMD with anti-VEGF injections have been published. Chandra et al. presented the results of a single-center (Moorfields Eye Hospital, London, UK), long-term 5-year wAMD therapy with aflibercept [19]; 468 patients (512 eyes) were included in the study, and 66% of them completed the fifth year. At the end of the follow-up period, there was a change in BCVA of −2.9 (SD 23.4) ETDRS letters, with a cumulative mean number of injections of 24.2. Chandra et al.’s results indicate significantly better long-term functional outcomes of wAMD treatment with systematic injections of aflibercept without numerical limitations.

Brynskov et al. presented 10-year results of wAMD therapy in a group of 3668 patients with newly diagnosed wAMD [20]. In the first year, on average, 5.4 intravitreal anti-VEGF injections (aflibercept, ranibizumab) were performed, and in the following years, 4.0–4.3 injections were administered. After 10 years, the researchers noted a mean BVCA deterioration within 5.0 (± 2.2) ETDRS letters, which in practice means many years of functional stabilization. For comparison with our observation, after the first year they found a change in BCVA by +0.7 ETDRS letters, after the second year −1.0, and after the third year −1.8 ETDRS letters.

Chandra et al. extended the analysis of the Moorfields Eye Hospital database to 10 years [21]. The mean BCVA change was −2.1 ETDRS letters. Over the course of 10 years, an average of 52.2 (SD 18.1) injections were performed; 67.1% of the subjects had a loss of less than 15 ETDRS letters, which was considered long-term functional stabilization, resulting from regular monitoring and treatment. Low baseline BCVA, foveola atrophy, and final size of atrophy area had a significant negative effect on final functional outcomes. In the Polish TP, in the following years, the analysis should be extended to include a thorough morphological assessment of atrophy and scarring, and these changes should be correlated with long-term functional outcomes.

As already mentioned, the treatment of wAMD in the Polish TP, apart from the first year of treatment with aflibercept, is based on the PRN regimen. However, the treat-and-extend (T&E) regimen is becoming more and more common globally, especially for aflibercept [22,23,24]. Researchers have shown that the T&E aflibercept regimen produces good results in routine practice, comparable to those obtained in randomized trials, with a significant reduction in both the number of follow-up visits and drug administrations over time. The high number of visits in the Polish TP results from the program requirements, which indicate the need for evaluation no less frequently than every 62 days. The results of Traine et al.’s study [24] can be used in the future to modify the Polish TP to a T&E regimen, which relieves the system of an excessive number of follow-up visits and allows for effective treatment with a number of injections comparable to our observation.

Our study included a large group of patients treated in various centers. On the one hand, this expands the study material, but on the other, it brings with it limitations and difficulties. The results were influenced by the reporting of data to the TPMS by various researchers, often using different methodologies, e.g., concerning functional parameters assessment or OCT. Because the treatment of wAMD in Poland was not systematized before the introduction of the TP, the methodology of dividing patients into subgroups of treatment-naïve and continuing-treatment was adopted. In this respect, the methodology of our observation is similar to the PERSEUS study. In the author’s opinion, it allows for a detailed analysis of the structure of the population treated in the TP. As shown by our results and the experience of other researchers, the adopted methodology allows us to demonstrate the importance of early wAMD therapy and its regularity, which in practice may be more important than the number of injections itself. The limitation of our observation is the strict description of the TP, limiting treatment to fixed or PRN regimens, and indicating the need for follow-up examinations no less frequently than every 62 days. This results in a high number of control tests, especially in the first year. However, taking into account other, previous studies from everyday practice, such as AURA or LUMINOUS, compliance with the regimen of control examinations and drug dosing in the treatment of wAMD allows for significantly better, stable functional effects [25,26]. If PRN regimen is selected for treatment wAMD patients, regular and planned evaluation of functional and morphological parameters without limitations of number necessary anti-VEGF injections, produces satisfactory treatment results (similar to the presented the Polish treatment program).

The wet AMD treatment program is being continued. Modifications of the exclusion criteria from 2020 indicate targeting PL towards injections, i.e., active treatment with limited follow-up examinations. wAMD is a chronic disease, and in consequence, the authors of this manuscript plan to include subsequent years in TP assessment.

## 4. Conclusions

Regular treatment of exudative age-related macular degeneration as part of the treatment program achieves functional stabilization and significant morphological improvement over long-term follow-up, with significantly fewer injections after the first year of treatment.

## Figures and Tables

**Table 1:** Patients’ baseline characteristics.

Variable	Category	Total	Therapy: Continuation	Therapy: Naive	*p*-Value
No. eyes		1430	626	804	
Age (years) (median (IQR))		78.0 (71.0, 83.0)	78.0 (71.0, 83.0)	78.0 (71.0, 83.0)	0.592
Sex (%)	Men	512 (0.36)	238 (0.38)	274 (0.34)	0.133
	Women	918 (0.64)	388 (0.62)	530 (0.66)	
Treatment (%)	Ranibizumab	347 (0.24)	137 (0.22)	210 (0.26)	0.062
	Aflibercept	1077 (0.75)	488 (0.78)	589 (0.73)	
Baseline BCVA, ETDRS letters; (median (IQR))		65.10 (50.05, 73.91)	65.10 (50.05, 69.95)	65.10 (50.05, 73.91)	**<0.05 ª**
Baseline BCVA (%); ETDRS letters	≤ 35	123 (0.09)	62 (0.10)	61 (0.08)	**<0.05**
	(35, 70)	936 (0.65)	421 (0.67)	515 (0.64)	
	>70	371 (0.26)	143 (0.23)	228 (0.28)	
Baseline BCVA, Snellen chart; (median (IQR))		0.4 (0.2, 0.6)	0.4 (0.2, 0.5)	0.4 (0.2, 0.6)	**<0.01 ª**
Baseline leakage area (%); (median (IQR))		70.0 (60.0, 80.0)	70.0 (60.0, 80.0)	70.0 (60.0, 80.0)	0.641
Baseline central retinal thickness (µm); (median (IQR))		344.0 (279.0, 419.75)	344.0 (276.0, 412.75)	343.5 (280.75, 420.75)	0.323
Baseline central retinal thickness; µm	≤200	77 (0.05)	35 (0.06)	42 (0.05)	0.483
	(200, 400)	930 (0.65)	416 (0.66)	514 (0.64)	
	>400	421 (0.29)	174 (0.28)	247 (0.31)	
Form of neovascularization	classic	171 (0.12)	63 (0.37)	108 (0.63)	0.050
	mixed	653 (0.46)	305 (0.47)	348 (0.53)	
	occult	606 (0.42)	258 (0.43)	348 (0.57)	

IQR: interquartile range; BCVA: best corrected visual acuity; ETDRS: Early Treatment of Diabetic Retinopathy Study. Treatment-naïve group includes eyes that were not treated before inclusion in the Polish Treatment Program. Continuation group includes eyes that were treated earlier and continued therapy within the Polish Treatment Program. All eyes include treatment-naïve and continuation. Presented *p*-values are testing for differences between the treatment-naïve group and those continuing treatment. The significance level is 0.05. The bold values indicate a statistically significant difference. ª When comparing a variable as a continuous variable, due to the large sample size, the differences identified as statistically significant do not have to be clinically relevant.

**Table 2:** Patients’ functional outcomes.

Variable	Year	Category	Total	Therapy: Continuation	Therapy: Naive	*p*-Value
No. eyes	baseline		1430	626	804	
	1		1430	626	804	
	2		1215	533	682	
	3		992	441	551	
BCVA, ETDRS letters (median (IQR))	baseline		65.10 (50.05, 73.91)	65.10 (50.05, 69.95)	65.10 (50.05, 73.91)	**<0.05 ª**
	1		65.10 (58.86, 74.79)	65.10 (50.05, 73.91)	65.10 (58.86, 74.79)	**<0.001 ª**
p vs. 1st entry			**<0.001 ª**	**<0.005 ª**	**<0.001 ª**	
	2		65.10 (60.26, 74.79)	65.10 (54.90, 74.79)	65.10 (60.26, 74.79)	**<0.05 ª**
p vs. 1st entry			**<0.05 ª**	0.249	**<0.05 ª**	
	3		65.10 (54.90, 74.79)	65.10 (54.90, 74.79)	65.10 (54.90, 74.79)	0.369
p vs. 1st entry			0.703	0.772	0.801	
Δ BCVA, ETDRS letters (mean (SD))	1		2.03 (12.15)	1.45 (12.04)	2.49 (12.22)	0.110
	2		0.94 (13.72)	0.69 (13.76)	1.13 (13.69)	0.580
p vs. 1st entry			**<0.001**	**<0.001**	**<0.001**	
	3		0.17 (14.05)	0.19 (13.81)	0.15 (14.24)	0.967
p vs. 1st entry			**<0.001**	**<0.001**	**<0.001**	
Baseline BCVA (%); ETDRS letters	baseline	≤35	123 (0.09)	62 (0.10)	61 (0.08)	**<0.05**
	baseline	(35, 70)	936 (0.65)	421 (0.67)	515 (0.64)	
	baseline	>70	371 (0.26)	143 (0.23)	228 (0.28)	
BCVA (%); ETDRS letters	1	≤35	59 (0.04)	38 (0.06)	21 (0.03)	**<0.001**
	1	(35, 70)	938 (0.66)	429 (0.69)	509 (0.63)	
	1	>70	426 (0.30)	158 (0.25)	268 (0.33)	
	2	≤35	27 (0.02)	14 (0.03)	13 (0.02)	0.098
	2	(35, 70)	822 (0.68)	375 (0.70)	447 (0.66)	
	2	>70	358 (0.29)	141 (0.26)	217 (0.32)	
	3	≤35	22 (0.02)	11 (0.02)	11 (0.02)	0.306
	3	(35, 70)	677 (0.68)	311 (0.71)	366 (0.66)	
	3	>70	284 (0.29)	116 (0.26)	168 (0.30)	
Baseline BCVA (Snellen chart); (median (IQR))	baseline		0.4 (0.2, 0.6)	0.4 (0.2, 0.50)	0.4 (0.2, 0.6)	**<0.01 ª**
BCVA (Snellen chart); (median (IQR))	1		0.4 (0.3, 0.625)	0.4 (0.2, 0.60)	0.4 (0.3, 0.625)	**<0.001 ª**
p vs. 1st entry			**<0.001 ª**	**<0.001 ª**	**<0.001 ª**	
	2		0.4 (0.32, 0.625)	0.4 (0.25, 0.63)	0.4 (0.32, 0.625)	**<0.05 ª**
p vs. 1st entry			**<0.005 ª**	0.090	**<0.05 ª**	
	3		0.4 (0.25, 0.625)	0.4 (0.25, 0.63)	0.4 (0.25, 0.625)	0.214
p vs. 1st entry			0.436	0.742	0.462	
Δ BCVA (Snellen chart) (median (IQR))	1		0.00 (−0.10, 0.13)	0.00 (−0.10, 0.10)	0.00 (−0.10, 0.20)	0.076
	2		0.00 (−0.10, 0.13)	0.00 (−0.10, 0.12)	0.00 (−0.10, 0.13)	0.620
p vs. 1st entry			**<0.001 ᵇ**	**<0.005 ᵇ**	**<0.001 ᵇ**	
	3		0.00 (−0.10, 0.13)	0.00 (−0.10, 0.12)	0.00 (−0.18, 0.20)	0.735
p vs. 1st entry			**<0.001 ᵇ**	**<0.001 ᵇ**	**<0.001 ᵇ**	

IQR: interquartile range; BCVA: best corrected visual acuity; ETDRS: Early Treatment of Diabetic Retinopathy Study. Treatment-naïve group includes eyes that were not treated before inclusion in the Polish Treatment Program. Continuation group includes eyes that were treated earlier and continued therapy within the Polish Treatment Program. All eyes include treatment-naïve and continuation. Presented *p*-values are testing for differences between the treatment-naïve group and those continuing treatment. The significance level is 0.05. The bold values indicate a statistically significant difference. ª When comparing a variable as a continuous variable, due to the large sample size, the differences identified as statistically significant do not have to be clinically relevant. ᵇ Baseline BCVA, final BCVA, and delta of BCVA are not normally distributed, thus medians (IQRs) are reported as descriptive statistics; difference of medians is generally not equal to median difference. Estimated 95% CI for median was used.

**Table 3:** Patients’ morphological outcomes.

Variable	Year	Category	Total	Therapy: Continuation	Therapy: Naive	*p*-Value
No. eyes	baseline		1430	626	804	
	1		1430	626	804	
	2		1215	533	682	
	3		992	441	551	
Central retinal thickness (um); (median (IQR))	baseline		344.00 (279.00, 419.75)	344.00 (276.00, 412.75)	343.50 (280.75, 420.75)	0.323
	1		269.00 (221.00, 330.25)	275.00 (223.00, 341.00)	261.00 (219.00, 323.00)	**<0.05**
p vs. 1st entry			**<0.001**	**<0.001**	**<0.001**	
	2		270.00 (221.00, 332.00)	270.00 (220.00, 336.00)	268.50 (222.00, 327.00)	0.773
p vs. 1st entry			**<0.001**	**<0.001**	**<0.001**	
	3		269.00 (219.00, 332.50)	267.50 (215.00, 331.25)	271.00 (220.00, 333.00)	0.772
p vs. 1st entry			**<0.001**	**<0.001**	**<0.001**	
Δ Central retinal thickness (um);(median (IQR))	1		−60.00 (−131.00, −4.00)	−49.00 (−119.00, 1.00)	−68.00 (−141.50, −7.00)	**<0.005**
	2		−55.00 (−133.50, 6.00)	−55.00 (−127.00, 12.50)	−56.00 (−142.00, 2.00)	0.441
p vs. 1st entry			**<0.001**	**<0.001**	**<0.001**	
	3		−65.00 (−142.50, 7.00)	−64.00 (−140.00, 13.50)	−68.00 (−143.00, 1.00)	0.763
p vs. 1st entry			**<0.001**	**<0.001**	**<0.001 ª**	
Injections; (median (IQR))	1		7.00 (5.00, 8.00)	5.00 (3.00, 7.00)	7.00 (7.00, 8.00)	**<0.001**
Injections; (median (IQR))	2		4.00 (2.00, 5.00)	4.00 (2.00, 5.00)	4.00 (2.00, 5.00)	0.857
p vs. 1st entry			**<0.001**	**<0.001**	**<0.001**	
Injections; (median (IQR))	3		4.00 (2.00, 5.00)	4.00 (2.00, 5.00)	4.00 (2.00, 5.00)	0.857
p vs. 1st entry			**<0.001**	**<0.001**	**<0.001**	
Visits; (median (IQR))	1		9.00 (9.00, 10.00)	9.00 (8.00, 9.00)	9.00 (9.00, 10.00)	**<0.001 ª**
Visits; (median (IQR))	2		7.00 (6.00, 8.00)	7.00 (6.00, 8.00)	7.00 (6.00, 8.00)	0.428
p vs. 1st entry			**<0.001**	**<0.001**	**<0.001**	
Visits; (median (IQR))	3		7.00 (6.00, 8.00)	7.00 (6.00, 8.00)	7.00 (6.00, 8.00)	0.428
p vs. 1st entry			**<0.001**	**<0.001**	**<0.001**	

IQR: interquartile range. Treatment-naïve group includes eyes that were not treated before inclusion in the Polish Treatment Program. Continuation group includes eyes that were treated earlier and continued therapy within the Polish Treatment Program. All eyes include treatment-naïve and continuation. Presented *p*-values are testing for differences between the treatment-naïve group and those continuing treatment. The significance level is 0.05. The bold values indicate a statistically significant difference. ª When comparing a variable as a continuous variable, due to the large sample size, the differences identified as statistically significant do not have to be clinically relevant.

## Data Availability

Therapeutic Program Monitoring System by National Health Fund.
